# Socially Learned Attitude Change is not reduced in Medicated Patients with Schizophrenia

**DOI:** 10.1038/s41598-018-37250-x

**Published:** 2019-01-30

**Authors:** Arndis Simonsen, Riccardo Fusaroli, Joshua Charles Skewes, Andreas Roepstorff, Ole Mors, Vibeke Bliksted, Daniel Campbell-Meiklejohn

**Affiliations:** 1 Psychosis Research Unit, Aarhus University Hospital, Risskov, Aarhus Denmark; 2 The Lundbeck Foundation Initiative for Integrative Psychiatric Research, iPSYCH, Aarhus, Denmark; 3 The Interacting Minds Centre, School of Culture and Society, Aarhus University, Aarhus, Denmark; 4 Center of Functionally Integrative Neuroscience, Department of Clinical Medicine, Aarhus University Hospital, Aarhus, Denmark; 5 The Psychiatric Centre, National Hospital of the Faroe Islands, Tórshavn, Faroe Islands; 6 Ílegusavnið, The Genetic Biobank of the Faroe Islands, Tórshavn, Faroe Islands; 7 School of Psychology, University of Sussex, Falmer, United Kingdom; 8 Cognitive Science, School of Communication and Culture, Aarhus University, Aarhus, Denmark

## Abstract

Schizophrenia is often associated with distinctive or odd social behaviours. Previous work suggests this could be due to a general reduction in conformity; however, this work only assessed the tendency to publicly agree with others, which may involve a number of different mechanisms. In this study, we specifically investigated whether patients display a reduced tendency to adopt other people’s opinions (socially learned attitude change). We administered a computerized conformity task, assumed to rely on reinforcement learning circuits, to 32 patients with schizophrenia or schizo-affective disorder and 39 matched controls. Each participant rated 153 faces for trustworthiness. After each rating, they were immediately shown the opinion of a group. After approximately 1 hour, participants were unexpectedly asked to rate all the faces again. We compared the degree of attitude change towards group opinion in patients and controls. Patients presented equal or more social influence on attitudes than controls. This effect may have been medication induced, as increased conformity was seen with higher antipsychotic dose. The results suggest that there is not a general decline in conformity in medicated patients with schizophrenia and that previous findings of reduced conformity are likely related to mechanisms other than reinforcement based social influence on attitudes.

## Introduction

Schizophrenia is often associated with distinctive or odd social behaviours. Already in the 1930s it was suggested that this could be due to a decline in responsiveness to social stimuli and studies from the 1960s and 70 s partly confirmed this hypothesis, indicating that patients with schizophrenia show reduced conformity to the expressed opinions of others^1–4^. Conformity refers to the act of changing one’s behaviour, opinions or beliefs to match or become more similar to those of others^5,6^.

The studies on conformity in schizophrenia were designed to measure public conformity, i.e. the tendency to deliberately and outwardly adopt others’ responses often in spite of clear contradictions to one’s own beliefs and without changing one’s belief or attitude^7^. This has also been referred to as public compliance^8,9^ and sheds light on social behaviour under social pressure.

It is unclear whether reduced conformity in patients with schizophrenia is restricted to public conformity or whether they display a general reduction in the tendency to conform to others, including a reduced tendency to adopt and maintain others’ behaviours, attitudes and beliefs in the absence of experienced social pressure. This has been termed private conformity or private acceptance and results in an internalization of others’ beliefs or preferences, i.e. a genuine change in beliefs^7,9^.

The aim of the current study was to investigate whether attitude change towards group opinion (private conformity) is altered in patients with schizophrenia (30/32 medicated) compared to healthy individuals. We used an automatic conformity paradigm informed by cognitive neuroscience^10,11^. In this computerized task, participants rate 153 faces for trustworthiness followed immediately by a group rating of the face. Approximately 1 hour later, participants are unexpectedly asked to rate all faces again in random order without receiving any information about the group ratings. The advantage of this setup is that there is no immediate social pressure to conform and original beliefs as well as change are measured. In addition, the high number of ratings make it unlikely that group ratings, seen during the first session, would be consciously remembered in the second session. Therefore, conformity in this task likely reflects an immediate, socially learned, change of attitude toward the group opinion^12,13^ and not simply public conformity. We consider this process to be relatively automatic, since participants are specifically asked to give their opinion and there is no task requirement or obvious reason to learn from the feedback as they cannot act on the feedback provided during the first round. When they are unexpectedly asked to rate the faces again 1 hour later, they presumably cannot deliberately act on the feedback given during the first round because they cannot consciously remember it.

Although few imaging studies have directly compared the neural mechanisms underlying public and private conformity^14^, the literature suggests that these are at least partially distinct. For instance, actual changes in opinion (value) or memory result in altered activity in orbitofrontal cortex (OFC)^13^ or in amygdala/hippocampus^14^, respectively, while public conformity does not result in such changes^14,15^. On the other hand, executive decisions to conform despite privately disagreeing, and thus inhibiting or overriding one’s current beliefs (public conformity), are assumed to rely more on prefrontal structures related to executive decision-making^16^. However, the evidence for this is still sparse, presumably due to lack of studies. It is thus entirely possible that patients would display impairments in one domain (public conformity) but not the other (private conformity) depending on the differential recruitment of brain structures and how these are affected in schizophrenia. For instance, it is well known that patients with schizophrenia display impairments in executive function and cognitive inhibition^17,18^. On the other hand, medicated patients display relatively intact learning from negative feedback in non-social contexts (e.g. avoidance of monetary loss)^19–23^ when it involves implicit or automatic learning processes. Such learning requires one to associate a low outcome value to a certain action. However, it is less clear whether the ability to represent *precise* expected values is intact in schizophrenia^21,24,25^.

These latter findings may be particularly relevant for this study, as we are measuring automatic updating of opinions (change in value) based on learning about group disagreement. Thus, in case a participant conforms, the task requires him/her not only to learn that certain actions are less favourable than expected (others disagree with him/her) but also to attribute a relatively precise updated value to the specific face, e.g. this face should be rated higher (and not lower) than initially thought. Indeed, imaging studies using this setup suggest that private conformity to group opinion is based on similar neural mechanisms as reinforcement learning^10,26,27^. Specifically, disagreement or conflict with group opinion can be seen as negative feedback that triggers deactivation of the ventral striatum and activation of the anterior insula (AI) and the dorsal part of the posterior medial frontal cortex (pMFC). This signals the discrepancy between preferred (agreement) and actual outcome (disagreement) thereby calling for an adjustment of the behaviour, i.e. an attitude change toward group opinion^10,26^ (when it is desirable to be similar to the group^28^). The final result is an update in OFC of the opinion or value assigned to the specific face, in this case^13^. The pMFC seems crucial in this conformity process^27^ as the size of the error related signal in pMFC predicts subsequent adjustments toward group opinion^10,29^, while down-regulation of the pMFC evoked by repetitive transcranial magnetic stimulation reduces such behavior^11^.

Interestingly, medicated patients show relatively intact error signals to negative feedback^30–32^. This feedback-related negativity signal (FRN) is also seen when receiving feedback about disagreement in conformity tasks, and is thought to correspond to the error-related signal detected in pMFC with fMRI^26,33,34^. Of course, the caveats of reverse inference need to be kept in mind here. For instance, the pMFC has been implicated in a variety of cognitive processes such as monetary loss, reduced reward, response conflict, negative feedback, physical and social pain, and error detection. It is therefore difficult to pinpoint the exact psychological process; however, it is generally assumed that it plays an important role in detecting (negative) changes in the environment that entail a subsequent adjustment of behavior^9,28^. Due to this very reason, the structures most often implicated in conformity (VS, AI, pMFC) have been proposed to convey general prediction errors (reinforcement learning)^10^, cognitive inconsistency^28^ and/or physical arousal and negative affective states^15^ that guide subsequent conformity. It is important to note that these are not mutually exclusive explanations^27^.

Another issue to keep in mind is that studies within pharmacology^35^ and genetics^36^, suggest that dopamine plays a role in private conformity to others’ opinion. This is potentially relevant for two reasons. First, schizophrenia is associated with altered dopamine function. Specifically, the acute psychotic state is associated with increased dopamine synthesis, release and resting-state dopamine concentrations^37^. Second, most patients receive some type of antipsychotic medication. These consistently block dopamine receptors^37^. Both of these factors could therefore potentially contribute to altered private conformity to others’ opinion in schizophrenia. Although it is difficult to predict the net result of these opposing factors, we know from studies on reinforcement learning, that both negative feedback learning and the associated FRN may increase with antipsychotic treatment^38^ and that patients’ sensitivity to negative feedback is positively associated with antipsychotic medication dose^39,40^. To the extent that parallels can be drawn between negative feedback learning and conformity to others’ opinions, these findings – together with the finding of relatively intact negative feedback learning in medicated patients – suggest that antipsychotic medication could play a role in how much one conforms to others’ opinion and that medicated patients could display relatively intact conformity. This of course requires that they are able to represent the updated values of the faces rather precisely. As there are potential medication effects, we also assessed whether conformity increased with antipsychotic dose. It should be noted that since we primarily tested medicated patients (30/32), we can only detect any potential deficits experienced prior to medication onset insofar that these are still present to some extent after medication onset. Finally, we wanted to investigate whether the patients’ tendency to conform was associated with their level of functioning, suggesting a possible route to the characteristic social impairments in case conformity was reduced.

## Materials and Methods

### Participants

The sample included 40 patients with an ICD-10 DCR diagnosis of schizophrenia or schizoaffective disorder and 40 healthy controls. Diagnosis was confirmed using the Schedules for Clinical Assessment in Neuropsychiatry (SCAN)^41,42^. Controls were matched pairwise to the patients by age, gender, childhood residence, and (when possible) to commenced educational level and parental socioeconomic status (see Table 1).

**Table 1:** Demographic and Clinical Characteristics of Patients and Controls.

	Schizophrenia (n = 32)	Controls (n = 39)
Age, mean (SD)	38.0 (11.1)	39.2 (10.6)
No. of males:females	21:11	27:12
Educational level commenced^a^, mean (SD)	2.0 (0.7)	2.3 (0.7)
Years of education, mean (SD)	12.1 (2.6)	14.2 (3.1)
No. of high: middle parental SES^b^	12:20	13:26
Level of functioning (PSP), mean (SD)	59.0 (15.4)	85.8 (4.9)
Positive symptoms (SAPS)^c^, mean (SD)	4.6 (4.2)	—
Negative symptoms (SANS)^d^, mean (SD)	7.4 (4.5)	—
CPZ equivalent dose in mg, mean (SD)	686 (561)	—

^a^Educational level commenced divided into 4 levels: 1: primary school (up to 10 years of education), 2: secondary school/technical training, 3: bachelor program, 4: master program. ^b^Parental socioeconomic status (SES) was divided into 3 levels: where a high SES corresponds to one of the parents having a high education + at least a middle annual income (above 200.000 DKK) or a middle education + a high income (above 400.000 DDK), while a middle SES corresponds to a high education + low income (below 200.000 DDK), a middle education + middle income (200.000–400.000 DDK) or no education + at least a middle income. None of the parents had a low SES. ^c^SAPS score is the total score of the 4 items: global rating of severity of hallucinations, delusions, bizarre behavior and the global rating of positive formal thought disorder. ^d^SANS score is the total score of the 5 items: global rating of affective flattening, alogia, avolition-apathy, anhedonia-asociality and attention.

Patients were recruited through the Psychiatric Centre of the National Hospital of the Faroe Islands. Controls were contacted based on their age and gender and, if they fulfilled the inclusion criteria and matched a patient, were offered to participate in the study. All the participants were native speakers of Faroese between the age of 18 and 55. Exclusion criteria for all participants included: current psychoactive substance use disorders (except nicotine), a neurological or medical disorder that could affect brain functioning, severe head trauma and an estimated IQ below 70 based on prior history or testing. In addition, the controls were excluded if they took any psychotropic medication or if they or a first-degree relative had a history of severe mental disorder. History of mental disorder for controls was assessed with SCAN. The participants were screened for recent use of psychoactive substances (THC/cannabis, opiates, amphetamine, MDMA, benzodiazepines, cocaine) using urine sticks (NanoSticka® 200-32). Patients with a positive test were excluded unless they had a prescription for the positive drug (e.g. benzodiazepines). None of the controls had a positive test.

Two patients had to be excluded as they did not fulfil inclusion criteria. Seven participants (6 patients, 1 control) only performed the first round of the task due to various reasons: one control and one patient only gave extremely high/low ratings, respectively, resulting in unbalanced feedback (i.e. they did not receive the full range of feedback possible in the task and higher/lower feedback only occurred on less than 4% of the trials instead of approximately 1/3 - see below for detailed task description), one patient said they did not understand task instructions, one did not want to perform the task, and one produced no variation in the ratings (>99% of ratings were identical). Two patients did not have time to perform the second round of the task. Thirty-two patients and thirty-nine controls performed both rounds of the task.

### Medication

At the time of testing, twenty-five patients had been on a stable dose of antipsychotic medication for at least 3 weeks, while five had adjustments made. Two patients were not taking antipsychotic medication.

Although antipsychotics may target several receptors and neurotransmitter systems, modulation of the D_2_ receptor seems to be a necessary and sufficient condition to obtain antipsychotic effects^43^. Antipsychotic medication doses can be converted into a common scale (e.g. chlorpromazine equivalents) based on their clinical efficacy. Importantly this efficacy is closely and specifically related to the occupancy of the dopamine D_2_ receptor (60–80%) and not, for instance, the serotonin 5-HT_2A_ receptor or other dopamine receptors^44–46^. Drugs with a high dissociation constant like clozapine and quetiapine also reach similar levels of occupancy, although it drops faster than with typical antipsychotics^44,47^. We converted all antipsychotic doses to chlorpromazine (CPZ) equivalents^48,49^ (see Table 2 for details). Some patients also took other types of medication (see Table S1).

**Table 2:** Chlorpromazine (CPZ) 100 mg/day dose equivalency.

Antipsychotic medication	mg per day/injection	no. of patients^d^
**Oral**		
Amisulpride^c^	116.3	1
Aripiprazole^b^	4	7
Chlorprothixene^c^	83.3	2
Clozapine^c^	66.7	6
Olanzapine^b^	3	9
Quetiapine^b^	60	5
Zuclopenthixol^c^	8.3	3
**Depot** ^**a**^		
Paliperidone^b^	15	2
Perphenazine decanoate^c^	41.4	1
Risperidone^b^	10	3
Zuclopenthixol decanoate^c^	66.4	4

^a^Depot antipsychotics were first converted to oral equivalencies of the same drug using suggested equivalencies based on studies of oral to depot switch^77^ or manufacturer’s recommendation^78,79^ and then converted to CPZ equivalents (see note b and c). For perphenazine decanoate we used the average minimum effective dose of perphenazine decanoate^80^ and equated it with the lowest recommended target dose of oral perphenazine^49^ and then converted to CPZ equivalents (see note c). ^b^For the second-generation antipsychotics, we used Leucht *et al*.^48^ to convert to CPZ equivalents when possible except for clozapine where the conversion result was highly questionable. ^c^For other antipsychotics (including clozapine), we used Gardner *et al*.^49^ to convert to CPZ equivalents. ^d^Eighteen patients were taking one antipsychotic, 11 were taking two and one was taking three.

### General procedure

The conformity task was administered as part of a larger battery of cognitive tasks. Symptom severity and level of functioning were assessed with the Scale for the Assessment of Positive/Negative Symptoms (SAPS/SANS)^50,51^ and the Personal and Social Performance Scale (PSP)^52^, respectively (see Table 1). The work complied with the ethical standards of the relevant national committees. The study was approved by the national ethics committee of the Faroe Islands, Vísindasiðsemisnevndin, and reported to the data protection agency, Dátueftirlitið. The work was carried out in accordance with the Declaration of Helsinki and written informed consent was obtained from all participants after the procedure had been explained.

### The conformity task

**Figure 1:**
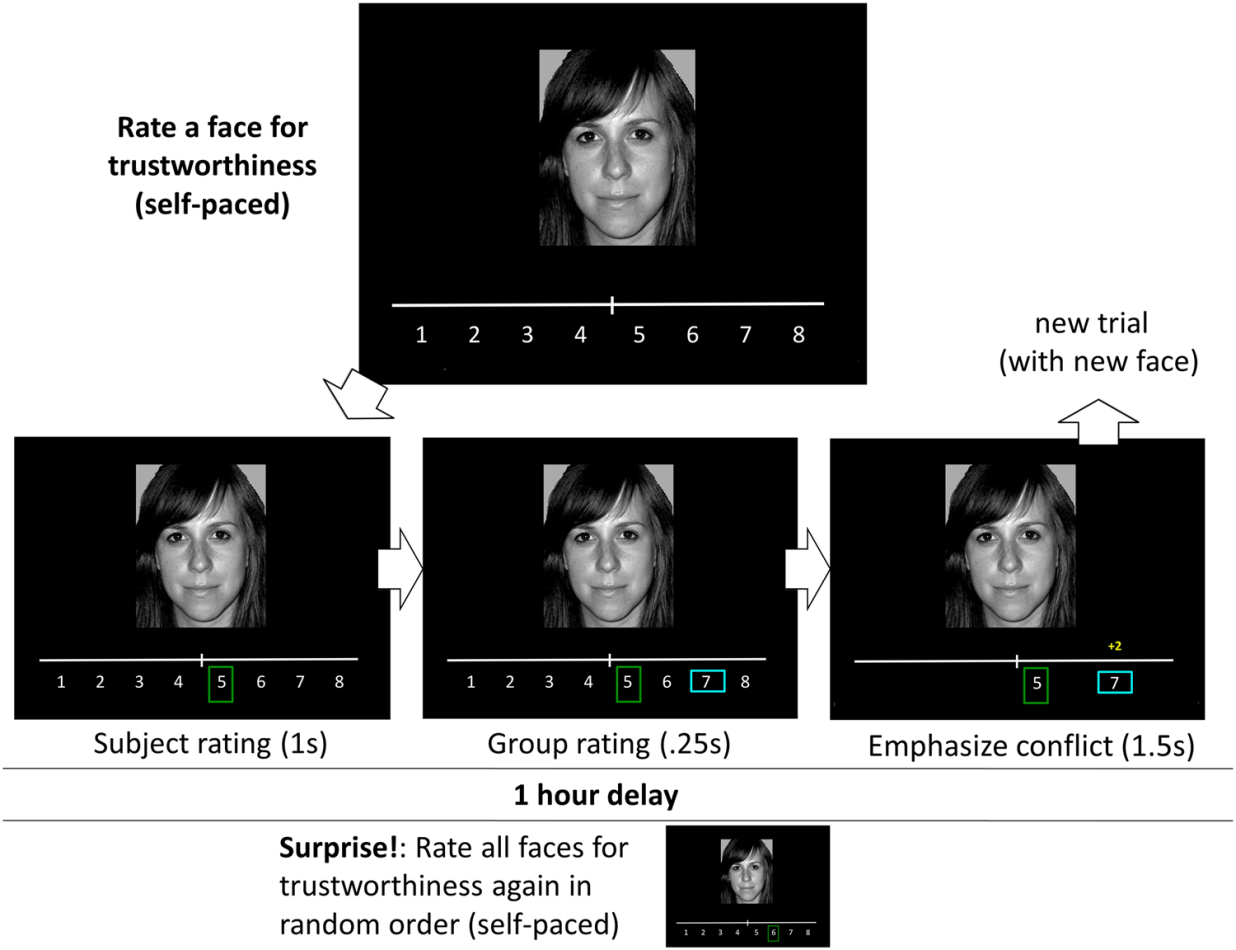
The conformity task, modified from Campbell-Meiklejohn *et al*.^35^. Participants rated 153 faces, one by one, for trustworthiness on a scale from 1 to 8. The choice was highlighted by a green vertical rectangle. After rating a face, participants learned the group opinion of that face, highlighted by a horizontal blue rectangle so that overlap with the participant’s response could be observed. Unexpectedly, participants rated the faces again after 1 hour, in a random order, without social feedback. Display was presented to participants in colour.

The conformity task was based on the task used in Klucharev *et al*.^10^ and was adapted from Campbell-Meiklejohn *et al*.^35^ and Simonsen *et al*.^53^. It consists of two rounds. On each trial of the first round, the participant rates a face for trustworthiness on an 8-point scale ranging from not at all trustworthy (1) to very trustworthy (8). Immediately after each rating, feedback of how other people rated the face (group opinion) is shown, as well as the difference between participant’s and the others’ rating (−3, −2, 0, 2, 3 points) (see Fig. 1). 153 randomized pictures of female faces were rated in total. After approximately 60 min. of solving other tasks, participants were asked to rate the faces again in a new random order. Participants were unaware that they had to rerate the faces until instructed to do so. No feedback on others’ ratings was given during the second round.

In order to provide an adequate number of trials in each condition, and minimize the number of participants required in the study, others’ ratings were generated pseudo-randomly by the computer with the following criteria: in approximately 1/3 of the trials, these ratings agreed with the participant’s rating, in approximately 1/3 ratings were two or three points lower than the participant’s, and in approximately 1/3 ratings were two or three points higher. The participants were told that the others’ ratings that matched their own rating within ±1 point would be shown as agreement (difference score of 0). The task was presented on a 19 in. computer screen using Presentation v. 16.3 (Neurobehavioral Systems).

### Statistical analysis

All analyses were set up as mixed effects (also called multilevel or hierarchical) regression models with changes in rating (second rating – first rating) as outcome, and feedback (−3, −2, 0, 2, 3), initial rating (1 to 8) and group (schizophrenia, controls) as fixed effects. Initial ratings were included to take any effects due to regression to the mean into account^54^. Variance inflation factors (VIF) were assessed in order to evaluate any issues with collinearity between initial rating and feedback. VIF provides an index of how much the variance of a coefficient is increased because of collinearity, with values of above 10 or even 4 often considered indicators of a potential problem^55^. Additionally, we ran an analysis including only ratings with well-matched feedback (i.e. only including initial ratings of 4 or 5, or initial ratings of 3 or 6 when feedback was −2, 0, or 2), to assess the robustness of the results. Finally, since both including initial ratings in the analysis or only analysing a subset of the data may provide conservative estimates of degree of conformity we reran the analysis on the whole dataset without including initial rating to assess a potential group difference in conformity.

There was not a pairwise match between all participants, however, data from the 32 patients and 39 controls were included in the analysis as the statistical methods employed are robust to moderate imbalance in the groups and, while preserving pairwise matching when present, they can rely on the additional data to reduce uncertainty in the estimates^56^. To account for the pairwise matching of participants, when present, matched individuals were assigned a common identifier, and entered as random intercept. As the one-to-one matching cannot be assumed to be homogeneous across all pairs, we included a random slope for group. This procedure explicitly estimates the non-independence introduced by matching and adjusts standard errors accordingly, which is a more conservative procedure than just assuming homogeneous matches. Analogously, we included random slopes for feedback and initial rating. Picture stimuli were also entered as random intercepts, and random slopes were included for feedback, initial rating and group.

When assessing the association between antipsychotic medication or level of functioning and conformity, the main analysis was run in patients only and the variable in question as well as potential confounders (e.g. symptom severity) were added as fixed effects, but otherwise maintaining the random structure of the full model. In order to investigate the robustness of a potential medication effect, we adjusted for a range of potential confounders: symptom severity, level of functioning, other medications or excluding patients with recent medication changes. In addition, we assessed whether we would see similar effects in patients receiving different types of antipsychotics. For instance, aripiprazole has a slightly different profile than the other antipsychotics. It is a functionally selective D_2_ ligand with both antagonistic and agonistic properties depending on the signalling environment of the D_2_ receptor^57^. We therefore looked at conformity effects within patients receiving aripiprazole vs. other antipsychotics. Similarly, we assessed whether effects were similar in patients receiving clozapine/quetiapine (high dissociation constant) vs. other antipsychotics (lower dissociation constant).

If a model could not converge, we simplified the random structure removing one random slope at a time until model estimation converged^58^. In case of null results on theoretically relevant questions, a follow up analysis was performed to assess the evidence in favour of the null hypothesis by estimating a Bayes factor (BF), which compares the relative likelihood of the null and of the alternative hypotheses given the observed data. If one hypothesis is more likely than the other given the data, there is evidence for that hypothesis over the other. The relative likelihood corresponds to relative evidence given the observed data, and is expressed as Bayes Factor. To calculate Bayes Factors we followed the procedure in Rouder and Morey (2012) for linear models with mixed effects (BayesFactor 0.9.12-2.) which employs Liang uninformative priors^59,60^, that is, a lack of prior expectations as to which hypothesis is better supported by the data. Bayes Factors below 1/3 were interpreted as substantial evidence in favour of the null hypothesis (more than 3 times as much evidence for the null hypothesis compared to the alternative hypothesis). Bayes Factors above three were interpreted as substantial evidence in favour of the alternative hypothesis (more than 3 times as much evidence for the alternative hypothesis compared to the null hypothesis). Values below 1 and approaching 1/3 favour the null over the alternative and vice versa, while values close to 1 do not present enough evidence to favour any of the hypotheses^61,62^.

All analyses were run using R 3.5^63^ and lme4 1.1-17^64^. Residuals were assessed with DHARMa^65^. Influential data points were checked with leave-one-out diagnostics^66^. To calculate p-values of the individual factors we treated the t-statistic as if it were a z-statistic (i.e., using a normal approximation), the most conservative estimation in large datasets^58^. The variance explained by the model was calculated using marginal R^2^ (R^2^m, including fixed factors only) and conditional R^2^ (R^2^c, full model including random structures), using MuMIn 1.40-4^67^. For model specifications and parameter estimates of the main analyses, see Tables S2 to S14 in the Supplementary Information.

## Results

### Ratings and feedback

In order to make sure that the task worked as expected in both groups, we assessed the mean and standard deviation of the ratings during the first and second round, as well as the number of times each type of feedback (lower, same, higher) was shown, see Table 3 for details. As can be seen from the table, the patients gave slightly lower and more variable ratings during both rounds compared to controls. The group difference was significant for average initial ratings (β = −0.31, SE = 0.14, t = −2.28, p = 0.03) but not for the second rating or the standard deviations (*p*s > 0.09). However, despite this group difference, both groups received a similar number of ratings for each type of feedback, and importantly also for feedback that was lower than the initial rating (*p*s > 0.5). The feedback was not perfectly balanced though: both groups received around 5-6 agreement trials more than anticipated, on average, and correspondingly fewer trials where group feedback was higher than the initial rating. However, since the feedback was so similar in the two groups, the computer-generated feedback was largely successful and this slight imbalance is unlikely to have affected the results.

**Table 3:** Ratings and Feedback.

	Schizophrenia (n = 32)	Controls (n = 39)
Initial rating, mean (SD)	4.7 (1.9)	5.0 (1.6)
Second rating, mean (SD)	4.8 (1.8)	5.0 (1.6)
No. of ratings with group giving lower feedback, mean (SD)	49.7 (2.62)	50.7 (0.88)
No. of ratings with agreement feedback, mean (SD)	56.6 (4.70)	55.8 (5.77)
No. of ratings with group giving higher feedback, mean (SD)	46.7 (5.21)	46.4 (5.91)

### Socially learned attitude change in schizophrenia

**Figure 2:**
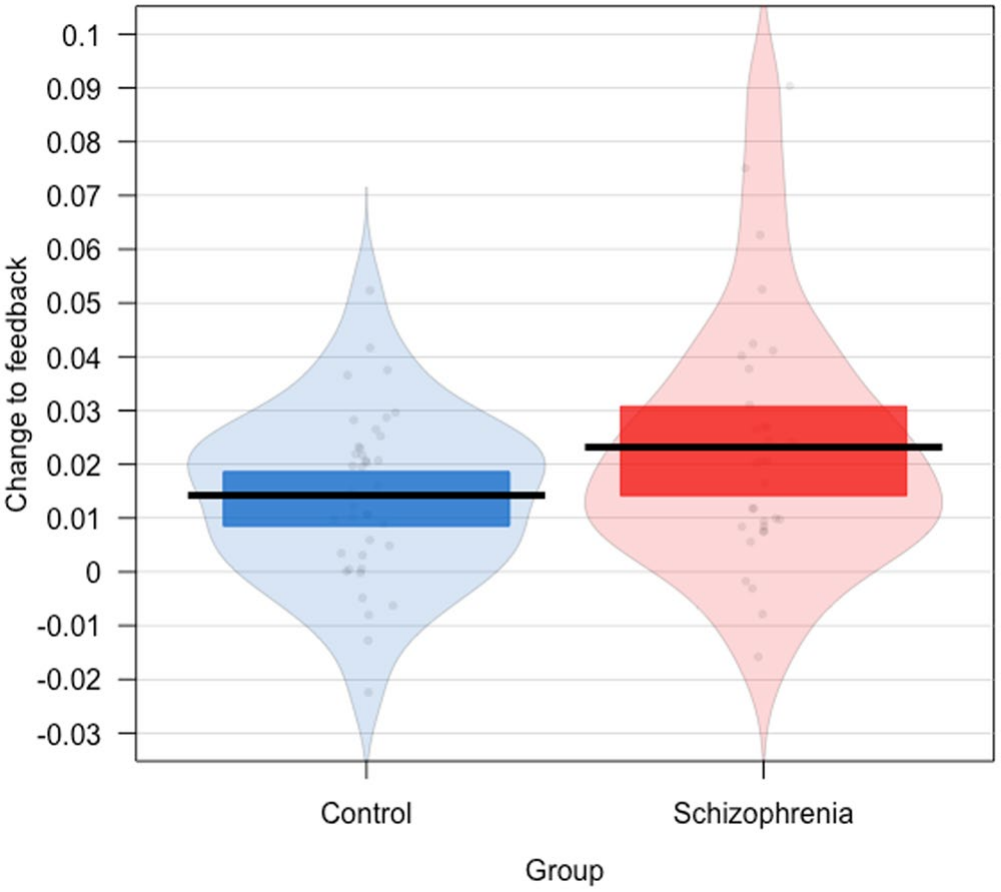
The effect of feedback on change in trustworthiness ratings in the two groups. Individual dots correspond to estimates for individual participants and were calculated as the participant-level random slope of feedback in a statistical model predicting change from feedback, group and initial ratings. The horizontal line is the mean for each group, the coloured bars indicate 95% Bayesian Credible Intervals of the mean and the background violin shapes, the distribution of the data.

As expected, there was a main effect of feedback (β = 0.018, SE = 0.008, t = 2.202, p = 0.03) and initial ratings (β = −0.663, SE = 0.021, t = −31.874, p < 0.001) on change of trustworthiness ratings. We did not observe a significant interaction between feedback and group (β = 0.008, SE = 0.013, t = 0.573, p = 0.567, R^2^m = 0.374, R^2^c = 0.496) (see Fig. 2) and the BF indicated substantial evidence of no difference in change to feedback in the two groups (BF = 0.023).

There was a correlation between initial rating and feedback due to the task design. However, the correlation was not very high (r = −0.396, SE = 0.009) when considering concerns of collinearity^68^. Consistent with this the variance inflation factors (VIFs) indicated no problem with the model (VIFs < 1.4). When running the analysis without initial rating on a subset of the data where initial ratings were matched across the different feedback conditions, the results were indeed highly similar to the above (main effect of feedback across groups: β = 0.030, SE = 0.009, t = 3.241, p = 0.001; interaction between feedback and group: β = −0.001, SE = 0.019, t = −0.052, p = 0.958, BF < 0.001). This latter approach effectively ensures no correlation between initial rating and feedback (r = 0.001, SE = 0.012), as well as avoids the confounder of regression to the mean with feedback, albeit it reduces the dataset from 153 to 99 data points per participant, on average.

Interestingly, when we adopted a less conservative procedure (i.e., analysing the whole dataset and not adjusting for initial ratings), patients did display significantly more conformity compared to controls (β = 0.053, SE = 0.016, t = 3.226, p = 0.001, R^2^m = 0.06, R^2^c = 0.08).

### Antipsychotic medication, level of functioning and attitude change

**Figure 3:**
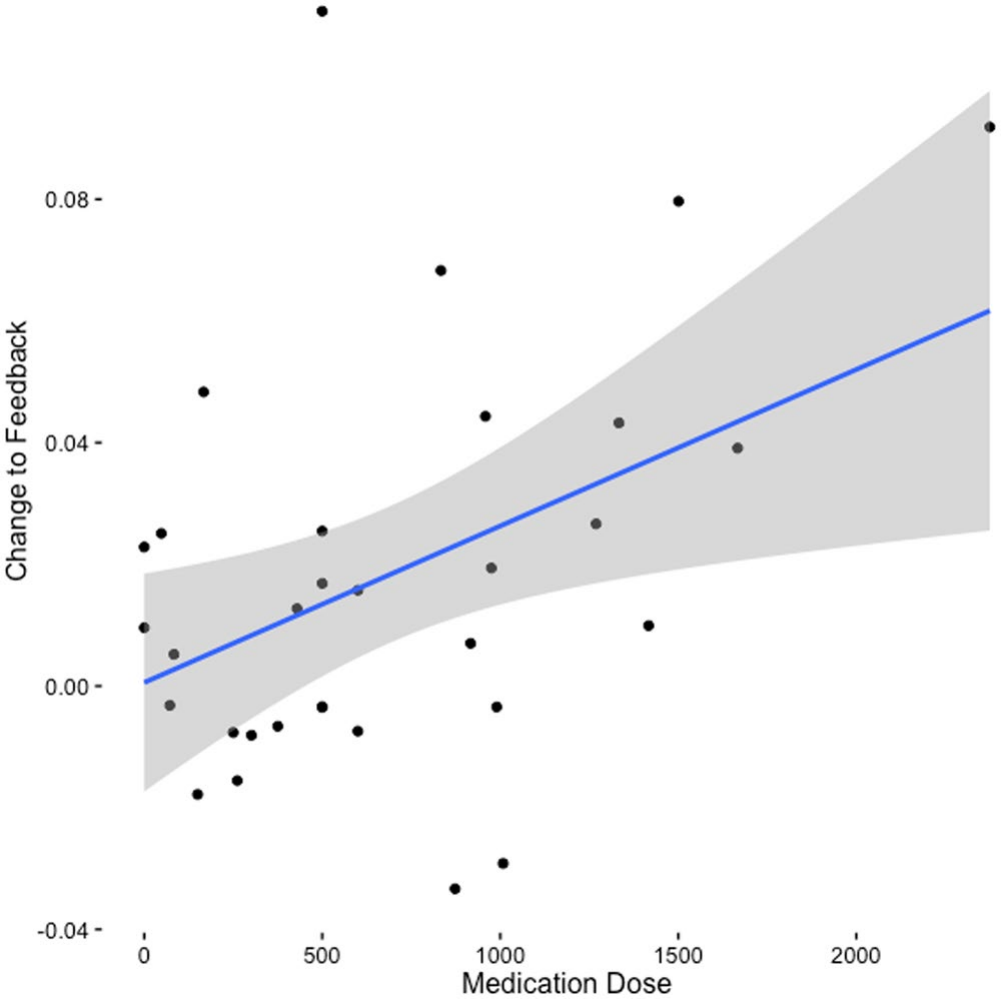
Relationship between antipsychotic medication dose and change to feedback as estimated for each individual patient. The shadow represents 95% confidence interval.

We observed an interaction between feedback and medication on change (for every 100 mg increase in CPZ: β = 0.006, SE = 0.002, t = 2.435, p = 0.021, R^2^m = 0.37, R^2^c = 0.491, see Fig. 3). This indicates that the higher the dose, the more the patient conformed to feedback. The interaction remained significant when adjusting for other types of medication (antidepressants, anticholinergics, proton-pump-inhibitors, hormonal contraceptives, benzodiazepines, corticosteroids, NSAID) (β = 0.011, SE = 0.003, t = 4.046, p < 0.001, R^2^m = 0.371, R^2^c = 0.487), negative symptom severity (β = 0.006, SE = 0.003, t = 2.266, p = 0.03, R^2^m = 0.371, R^2^c = 0.491), positive symptom severity (β = 0.008, SE = 0.003, t = 2.976, p = 0.005, R^2^m = 0.37, R^2^c = 0.488), level of functioning (β = 0.008, SE = 0.003, t = 3.068, p = 0.004, R^2^m = 0.37, R^2^c = 0.49), or when excluding the 5 patients with recent medication changes (β = 0.006, SE = 0.003, t = 2.323, p = 0.028, R^2^m = 0.364, R^2^c = 0.482). In these models there was not a significant interaction between feedback and negative symptom severity (β > −0.001, SE = 0.003, t = −0.036, p = 0.971, BF = 0.034) or positive symptom severity (β = −0.005, SE = 0.003, t = −1.599, p = 0.121, BF = 0.19) on change. PSP showed a statistical trend indicating higher conformity in patients with a higher level of functioning when adjusting for medication dose (β = 0.002, SE = 0.001, t = 1.772, p = 0.086).

Patients receiving aripiprazole (7 patients) and patients receiving other antipsychotics (23 patients) showed a similar interaction between feedback and dose on change (aripiprazole: β = 0.011, SE = 0.004, t = 2.506, p = 0.032, R^2^m = 0.323, R^2^c = 0.397; other antipsychotics: β = 0.006, SE = 0.003, t = 1.815, p = 0.083, R^2^m = 0.363, R^2^c = 0.482) and the BF indicated substantial evidence of no difference between these groups (BF = 0.054). It should be noted that 5 out of 7 patients receiving aripiprazole also received other types of antipsychotic medication. Similarly, patients receiving clozapine/quetiapine (11 patients) and patients receiving other antipsychotics (19 patients) showed a similar interaction between feedback and dose on change (clozapine/quetiapine: β = 0.012, SE = 0.004, t = 2.952, p = 0.003, R^2^m = 0.381, R^2^c = 0.489; other antipsychotics: β = 0.008, SE = 0.004, t = 2.244, p = 0.036, R^2^m = 0.34, R^2^c = 0.443) and the BF indicated substantial evidence of no difference between these groups (BF = 0.046). Four out of 11 patients receiving clozapine/quetiapine also received other types of antipsychotic medication.

## Discussion

We found that medicated patients with schizophrenia do not present a reduced tendency to conform to others’ opinions when there is minimal social pressure. In fact, patients displayed at least similar or even enhanced levels of attitude change towards group opinion compared to healthy individuals. The results suggest that patients with schizophrenia – at least when medicated – cannot be characterized as suffering from a general decline in conformity behaviour as previous literature would suggest^1–4^. Reduced conformity may be restricted to situations that require deliberate considerations as to whether to conform to social pressure or not (public conformity). Such situations may rely heavily on situational scripts and considerations about how to tactfully deal with situations in which there is conflict between being honest and being agreeable^69^. As such, they are assumed to rely more on prefrontal structures related to executive decision-making^16^. Consistent with this idea, patients display impairments in executive function and cognitive inhibition^17,18^.

Our finding is consistent with studies reporting intact negative feedback learning in reinforcement learning tasks^19–23^. This study places these important effects in a social context where updates to beliefs and behaviour happen rapidly within a single trial (as opposed to reinforcement learning over multiple trials). Indeed, the mechanism behind private conformity to others’ opinions has been proposed to rely on reinforcement learning circuits where disagreement or conflict with group opinion is seen as negative feedback that triggers a prediction error signal in the ventral striatum and pMFC^10,26^, resulting in an update in opinion, represented in OFC^13^.

It could be argued that a potentially enhanced tendency to conform to others’ opinions actually reflects a deficit. There are several factors that suggest that this was not the case. First, conformity was not associated with symptom severity. Second, there was a trend association between higher level of functioning and increased conformity. This is in the opposite direction to what would be expected, if increased conformity reflected a deficit and suggests that increased susceptibility to social influence is beneficial. Third, the degree of attitude change in patients was dose-dependent: the higher the antipsychotic dose, the more change towards group opinion was seen. This suggests that increased susceptibility to social influence may be medication induced. If this is indeed the case, then the treatment may mask a potential trait deficit in conformity or resolve a potential state deficit, and in theory, antipsychotic medication could render patients more susceptible to social influence compared to healthy individuals, as we see when not controlling for regression to the mean.

The association between medication dose and conformity remained significant when controlling for other medications, symptom severity and level of functioning and was similar in patients receiving different antipsychotics. The observed association is consistent with recent studies on schizophrenia showing increased sensitivity to negative feedback in non-social contexts with increasing antipsychotic medication dose^39,40^ and a normalization of response to negative feedback when on medication^38^. Similar medication effects are also seen in Tourette’s syndrome^70^. As conformity and negative feedback learning can be modulated pharmacologically in healthy individuals^35,53,71^, we expect the effect seen in this study to not be specific to schizophrenia but a general effect of modulating the neurotransmitter systems.

The mechanism behind this possible antipsychotic medication effect is not known; however, previous literature suggests that the enhanced conformity might be mediated by enhanced error signalling in pMFC to disagreement^10,11,27^ and antipsychotic treatment seems to enhance these error signals^38^. Frank *et al*.^71,72^ have put forward a neurocomputational model on how this may come about. Specifically, learning from positive and negative feedback is suggested to depend on differences in functioning of two distinct pathways in the basal ganglia (BG), where specifically negative feedback learning would rely on transient cessation of tonic dopamine cell firing (dopamine dips) causing release of inhibition of the indirect pathway and reduced activity in the direct pathway thereby suppressing inappropriate or unrewarding responses^71,72^. These prediction error signals originating in the BG are thought to reach the pMFC that acts as a control filter recruiting necessary adjustments when required and to modulate the pMFC activity thus resulting in larger error signals to unfavourable outcomes^73^. Importantly, the model predicts how dopamine modulation affects learning, e.g. D_2_ blockade with chronic antipsychotic administration should have a similar effect on the indirect pathway as dopamine dips resulting in increased learning from negative feedback^71,72^. The indirect pathway with its D_2_ receptors is thought to play a critical role in modulating the activity in pMFC resulting in a larger FRN or a stronger hemodynamic signal when an outcome is worse than expected^73^. Of course, it is not sufficient to increase the error signals in pMFC if patients have difficulties representing precise values (of e.g. faces) in OFC. However, our results suggest that patients are indeed able to represent the updated value attributed to the individual faces rather precisely, as they would not be able to conform otherwise, i.e. they would not know whether a rating “should be” higher or lower than initially assumed. Whether Frank *et al*.’s model extends to private conformity to others’ opinions would have to be formally tested. Alternatively, enhanced conformity may be mediated by antipsychotic medication induced alterations in oxytocin functioning^74^. Previous studies in healthy individuals suggest that oxytocin may enhance conformity^75,76^.

The study has some limitations. First, as most of the patients in this study were medicated, it is not clear whether unmedicated patients would display reduced conformity compared to controls. Future studies in drug-naïve patients could shed further light on this. Second, as patients were not randomized to different treatments, we cannot exclude that unmeasured individual differences accompanying medication dose contributed to the observed association between medication dose and conformity.

In summary, medicated patients with schizophrenia do not display a reduction in social influence on attitudes on a task shown to evoke conflict-driven attitude change within established reinforcement-learning systems. Social deficits and previous findings of residual non-conformity in schizophrenia are likely related to mechanisms other than reinforcement based social influence on attitudes. Intact susceptibility to social influence may be a result of antipsychotic treatment. Future studies can build on this finding to investigate further the mechanisms by which treatment may modulate social learning and explore other cognitive mechanisms of non-conformity in schizophrenia.

## Supplementary information

Supplementary Information

## Data Availability

The dataset analysed during the current study is available from the corresponding author on reasonable request.
