# Supplementary material for: Socially Learned Attitude Change is not reduced in Medicated Patients with Schizophrenia
Source: Sci Rep. 2019 Jan 30;9:992. doi: 10.1038/s41598-018-37250-x (PMC6353936; doi:10.1038/s41598-018-37250-x)
Supplement: Supplementary file 1 — Supplementary Information [file 41598_2018_37250_MOESM1_ESM.doc]

Supplementary Information

**Socially Learned Attitude Change is not reduced in Medicated Patients with Schizophrenia**

Arndis Simonsen, Riccardo Fusaroli, Joshua Charles Skewes, Andreas Roepstorff,

Ole Mors, Vibeke Bliksted, Daniel Campbell-Meiklejohn

| **Table S1** |  |
| --- | --- |
| **Medication type** | **no. of patients** |
| Antidepressant | 9 |
| Anticholinergic | 7 |
| Proton pump inhibitor | 4 |
| Corticosteroid | 4 |
| Hormonal contraceptive | 3 |
| NSAID | 3 |
| Benzodiazepine | 3 |
| Anticonvulsant | 2 |
| Melatonin | 2 |
| Antabus (Disulfiram) | 2 |
| Simvastatin | 2 |
| Anxiolytic non-BDZ | 1 |
| Lithium | 1 |
| Antibiotic | 1 |
| ACE inhibitor | 1 |
| Antidiabetic | 1 |
| Betablocker | 1 |
| Levothyroxine | 1 |
| Paracetamol | 1 |
| Opioid | 1 |

**Report of the full statistical estimates of models described in the manuscript**

*Table S2 – Main effects of feedback, group and initial rating on change. The model was defined (according to the lme4 software syntax) as: Change ~ 1 + Feedback + Group + Initial Rating + (1 + Feedback + Group + Initial Rating | ParticipantPair ID) + (1 + Feedback + Group + Initial Rating | Stimulus Picture).*

| **Factor** | **ß** | **SE** | **t** | **p** |
| --- | --- | --- | --- | --- |
| *Intercept* | 3.286 | 0.121 | 27.108 | <0.001 |
| *Feedback* | 0.018 | 0.008 | 2.202 | 0.03 |
| *Group* | -0.075 | 0.107 | -0.701 | 0.488 |
| *Initial Rating* | -0.663 | 0.021 | -31.874 | <0.001 |

*Table S3 – Interaction between feedback and group on change, adjusting for initial rating. The model was defined (according to the lme4 software syntax) as: Change ~ 1 + Feedback × Group + Initial Rating + (1 + Feedback + Group + Initial Rating | ParticipantPair ID) + (1 + Feedback + Group + Initial Rating | Stimulus Picture).*

| **Factor** | **ß** | **SE** | **t** | **p** |
| --- | --- | --- | --- | --- |
| Intercept | 3.285 | 0.121 | 27.089 | <0.001 |
| Feedback | 0.014 | 0.01 | 1.442 | 0.15 |
| Group | -0.073 | 0.107 | -0.685 | 0.499 |
| Initial Rating | -0.662 | 0.021 | -31.827 | <0.001 |
| Feedback × Group | 0.008 | 0.013 | 0.573 | 0.567 |

*Table S4 – Interaction between group and feedback on change (not adjusting for initial rating). The model was defined (according to the lme4 software syntax) as: Change ~ 1 + Feedback × Group + (1 + Group + Feedback | ParticipantPair ID) + (1 + Group + Feedback | Stimulus Picture).*

| **Factor** | **ß** | **SE** | **t** | **p** |
| --- | --- | --- | --- | --- |
| Intercept | -0.01 | 0.038 | -0.262 | 0.795 |
| Feedback | 0.20 | 0.014 | 13.938 | <0.001 |
| Group | 0.094 | 0.058 | 1.612 | 0.116 |
| Feedback × Group | 0.053 | 0.016 | 3.226 | 0.001 |

*Table S5 – Effects of feedback, initial rating and medication dose on change. The model was defined as: Change ~ 1 + Feedback × Medication Dose + Initial Rating + (1 + Feedback + Initial Rating | Participant ID) + (1 + Feedback + Initial Rating | Stimulus Picture).*

| **Factor** | **ß** | **SE** | **t** | **p** |
| --- | --- | --- | --- | --- |
| *Intercept* | 3.024 | 0.215 | 14.038 | <0.001 |
| *Feedback* | -0.018 | 0.023 | -0.794 | 0.433 |
| *Medication Dose* | 0.022 | 0.016 | 1.38 | 0.177 |
| *Initial Rating* | -0.654 | 0.029 | -22.629 | <0.001 |
| *Feedback × Dose* | 0.006 | 0.002 | 2.435 | 0.021 |

*Table S6 – Effects of feedback, initial rating and medication dose on change, when adjusting for other drugs (note: only medications taken by more than 2 patients were included in the analysis). The model was defined as: Change ~ 1 + Initial Rating + Feedback × (Medication Dose + Antidepressant + Proton Pump Inhibitor + Anticholinergic + Corticosteroid + Contraception + NSAID + Benzodiazepine) + (1 + Initial Rating + Feedback | Participant ID) + (1 + Initial Rating + Feedback | Stimulus Picture).*

| **Factor** | **ß** | **SE** | **t** | **p** |
| --- | --- | --- | --- | --- |
| *Intercept* | 3.045 | 0.241 | 12.624 | <0.001 |
| *Feedback* | -0.033 | 0.025 | -1.348 | 0.178 |
| *Medication Dose* | 0.019 | 0.019 | 1.023 | 0.314 |
| *Initial Rating* | -0.652 | 0.029 | -22.45 | <0.001 |
| *Antidepressant* | -0.147 | 0.207 | -0.709 | 0.483 |
| *Proton pump inhib.(ppi)* | 0.173 | 0.309 | 0.561 | 0.579 |
| *Anticholinergic* | 0.078 | 0.243 | 0.323 | 0.749 |
| *Corticosteroid* | -0.005 | 0.353 | -0.014 | 0.989 |
| *Contraceptive* | -0.197 | 0.323 | -0.611 | 0.545 |
| *NSAID* | -0.032 | 0.356 | -0.09 | 0.929 |
| *Benzodiazepine* | 0.138 | 0.386 | 0.357 | 0.724 |
| *Feedback × Dose* | 0.011 | 0.003 | 4.046 | <0.001 |
| *Feedback × Antidep.* | -0.029 | 0.026 | -1.106 | 0.27 |
| *Feedback × ppi* | -0.12 | 0.04 | -3.02 | 0.003 |
| *Feedback × Antichol.* | -0.053 | 0.03 | -1.739 | 0.083 |
| *Feedback × Corticoster.* | 0.075 | 0.045 | 1.693 | 0.091 |
| *Feedback × Contracept.* | 0.07 | 0.041 | 1.737 | 0.083 |
| *Feedback × NSAID* | 0.016 | 0.046 | 0.36 | 0.719 |
| *Feedback × Benzodiaz.* | 0.016 | 0.049 | 0.335 | 0.738 |

*Table S7 – Effects of feedback, initial rating and medication dose on change, when adjusting for negative symptom severity (SANS). The model was defined as Change ~ 1 + Initial Rating + Feedback × (Medication Dose + SANS) + (1 + Initial Rating + Feedback | Participant ID) + (1 + Initial Rating + Feedback | Stimulus Picture).*

| **Factor** | **ß** | **SE** | **t** | **p** |
| --- | --- | --- | --- | --- |
| *Intercept* | 2.94 | 0.244 | 12.065 | <0.001 |
| *Feedback* | -0.018 | 0.029 | -0.614 | 0.543 |
| *Medication Dose* | 0.018 | 0.017 | 1.036 | 0.308 |
| *Initial Rating* | -0.654 | 0.029 | -22.636 | <0.001 |
| *SANS* | 0.015 | 0.021 | 0.718 | 0.478 |
| *Feedback × Dose* | 0.006 | 0.003 | 2.266 | 0.03 |
| *Feedback × SANS* | >-0.001 | 0.003 | -0.036 | 0.971 |

*Table S8 – Effects of feedback, initial rating and medication dose on change, when adjusting for positive symptom severity (SAPS). The model was defined as Change ~ 1 + Initial Rating + Feedback × (Medication Dose + SAPS) + (1 + Initial Rating + Feedback | Participant ID) + (1 + Initial Rating + Feedback | Stimulus Picture).*

| **Factor** | **ß** | **SE** | **t** | **p** |
| --- | --- | --- | --- | --- |
| *Intercept* | 3.084 | 0.22 | 14.035 | <0.001 |
| *Feedback* | -0.006 | 0.024 | -0.246 | 0.807 |
| *Medication Dose* | 0.03 | 0.017 | 1.739 | 0.092 |
| *Initial Rating* | -0.655 | 0.029 | -22.651 | <0.001 |
| *SAPS* | -0.025 | 0.023 | -1.074 | 0.291 |
| *Feedback × Dose* | 0.008 | 0.003 | 2.976 | 0.005 |
| *Feedback × SAPS* | -0.005 | 0.003 | -1.599 | 0.121 |

*Table S9 – Effects of feedback, initial rating and medication dose on change, when adjusting for level of functioning (PSP). The model was defined as Change ~ 1 + Initial Rating + Feedback × (Medication Dose + PSP) + (1 + Initial Rating + Feedback | Participant ID) + (1 + Initial Rating + Feedback | Stimulus Picture).*

| **Factor** | **ß** | **SE** | **t** | **p** |
| --- | --- | --- | --- | --- |
| *Intercept* | 2.845 | 0.466 | 6.099 | <0.001 |
| *Feedback* | -0.125 | 0.064 | -1.953 | 0.059 |
| *Medication Dose* | 0.025 | 0.017 | 1.45 | 0.157 |
| *Initial Rating* | -0.655 | 0.029 | -22.652 | <0.001 |
| *PSP* | 0.003 | 0.006 | 0.439 | 0.664 |
| *Feedback × Dose* | 0.008 | 0.003 | 3.068 | 0.004 |
| *Feedback × PSP* | 0.002 | 0.001 | 1.772 | 0.086 |

*Table S10 – Effects of feedback, initial rating and medication dose on change, when excluding patients who had medication changes within the previous 3 weeks. Change ~ 1 + Initial Rating + Feedback × Medication Dose + (1 + Initial Rating + Feedback | Participant ID) + (1 + Initial Rating + Feedback | Stimulus Picture).*

| **Factor** | **ß** | **SE** | **t** | **p** |
| --- | --- | --- | --- | --- |
| *Intercept* | 2.86 | 0.223 | 12.812 | <0.001 |
| *Feedback* | -0.013 | 0.025 | -0.509 | 0.615 |
| *Medication Dose* | 0.026 | 0.017 | 1.558 | 0.131 |
| *Initial Rating* | -0.632 | 0.029 | -22.111 | <0.001 |
| *Feedback × Dose* | 0.006 | 0.003 | 2.323 | 0.028 |

*Table S11 – Effects of feedback, initial rating and medication dose on change, in patients receiving aripiprazole. Change ~ 1 + Initial Rating + Feedback ×* Medication Dose + (1 + Initial Rating + Feedback | Participant ID) + (1 + Feedback | Stimulus Picture).

| **Factor** | **ß** | **SE** | **t** | **p** |
| --- | --- | --- | --- | --- |
| *Intercept* | 2.397 | 0.343 | 6.984 | <0.001 |
| *Feedback* | -0.093 | 0.052 | -1.785 | 0.111 |
| *Medication Dose* | 0.058 | 0.014 | 4.259 | 0.002 |
| *Initial Rating* | -0.616 | 0.047 | -13.086 | <0.001 |
| *Feedback × Dose* | 0.011 | 0.004 | 2.506 | 0.032 |

*Table S12 – Effects of feedback, initial rating and medication dose on change, in patients receiving antipsychotics other than aripiprazole. Change ~ 1 + Initial Rating + Feedback ×* Medication Dose + (1 + Initial Rating + Feedback | Participant ID) + (1 + Feedback | Stimulus Picture).

| **Factor** | **ß** | **SE** | **t** | **p** |
| --- | --- | --- | --- | --- |
| *Intercept* | 3.139 | 0.273 | 11.507 | <0.001 |
| *Feedback* | -0.025 | 0.029 | -0.883 | 0.385 |
| *Medication Dose* | 0.006 | 0.024 | 0.236 | 0.816 |
| *Initial Rating* | -0.646 | 0.035 | -18.572 | <0.001 |
| *Feedback × Dose* | 0.006 | 0.003 | 1.815 | 0.083 |

*Table S13 – Effects of feedback, initial rating and medication dose on change, in patients receiving clozapine or quetiapine. Change ~ 1 + Initial Rating + Feedback ×* Medication Dose + (1 + Initial Rating + Feedback | Participant ID) + (1 + Feedback | Stimulus Picture).

| **Factor** | **ß** | **SE** | **t** | **p** |
| --- | --- | --- | --- | --- |
| *Intercept* | 2.746 | 0.365 | 7.519 | <0.001 |
| *Feedback* | -0.097 | 0.049 | -1.959 | 0.055 |
| *Medication Dose* | 0.048 | 0.03 | 1.598 | 0.139 |
| *Initial Rating* | -0.658 | 0.05 | -13.276 | <0.001 |
| *Feedback × Dose* | 0.012 | 0.004 | 2.952 | 0.003 |

*Table S14 – Effects of feedback, initial rating and medication dose on change, in patients receiving antipsychotics other than clozapine or quetiapine. Change ~ 1 + Initial Rating + Feedback ×* Medication Dose + (1 + Initial Rating + Feedback | Participant ID) + (1 + Feedback | Stimulus Picture).

| **Factor** | **ß** | **SE** | **t** | **p** |
| --- | --- | --- | --- | --- |
| *Intercept* | 2.929 | 0.265 | 11.056 | <0.001 |
| *Feedback* | -0.03 | 0.03 | -1.012 | 0.324 |
| *Medication Dose* | 0.021 | 0.018 | 1.174 | 0.257 |
| *Initial Rating* | -0.618 | 0.034 | -18.438 | <0.001 |
| *Feedback × Dose* | 0.008 | 0.004 | 2.244 | 0.036 |
